# Machine-learning analysis of intrinsically disordered proteins identifies key factors that contribute to neurodegeneration-related aggregation

**DOI:** 10.3389/fnagi.2022.938117

**Published:** 2022-08-03

**Authors:** Akshatha Ganne, Meenakshisundaram Balasubramaniam, Srinivas Ayyadevara, Robert J. Shmookler Reis

**Affiliations:** ^1^Bioinformatics Program, University of Arkansas for Medical Sciences and University of Arkansas at Little Rock, Little Rock, AR, United States; ^2^Department of Geriatrics, University of Arkansas for Medical Sciences, Little Rock, AR, United States; ^3^Central Arkansas Veterans Healthcare System, Little Rock, AR, United States

**Keywords:** proteostasis, misfolding and aggregation, Alzheimer’s disease, Parkinson’s disease, drug screening and discovery, intrinsically disordered proteins (IDPs), neural network, support vector machine

## Abstract

Protein structure is determined by the amino acid sequence and a variety of post-translational modifications, and provides the basis for physiological properties. Not all proteins in the proteome attain a stable conformation; roughly one third of human proteins are unstructured or contain intrinsically disordered regions exceeding 40% of their length. Proteins comprising or containing extensive unstructured regions are termed intrinsically disordered proteins (IDPs). IDPs are known to be overrepresented in protein aggregates of diverse neurodegenerative diseases. We evaluated the importance of disordered proteins in the nematode *Caenorhabditis elegans*, by RNAi-mediated knockdown of IDPs in disease-model strains that mimic aggregation associated with neurodegenerative pathologies. Not all disordered proteins are sequestered into aggregates, and most of the tested aggregate-protein IDPs contribute to important physiological functions such as stress resistance or reproduction. Despite decades of research, we still do not understand what properties of a disordered protein determine its entry into aggregates. We have employed machine-learning models to identify factors that predict whether a disordered protein is found in sarkosyl-insoluble aggregates isolated from neurodegenerative-disease brains (both AD and PD). Machine-learning predictions, coupled with principal component analysis (PCA), enabled us to identify the physiochemical properties that determine whether a disordered protein will be enriched in neuropathic aggregates.

## Introduction

Proteins play critical and significant roles in every regulatory network that governs an organism’s cellular and physiological functions. Protein folding is a critical step in achieving a functional state; each protein transitions under physiological conditions to attain the conformation with the lowest possible free energy (Rose et al., 2006). Proteins that never attain a stable folded conformation, and that lack rigid tertiary or quaternary three-dimensional structures, are termed intrinsically disordered proteins (IDPs). IDP conformations are not fixed by the thermodynamics of single native proteins, but are able to vary due to protein-protein or protein-ligand interactions, and/or as a result of post-translational modifications (PTMs). Nearly a third of all proteins in the human proteome have been classified as unstructured or intrinsically disordered proteins (Ali and Ivarsson, 2018; Deiana et al., 2019). These IDPs play important roles in multiple physiological processes such as vesicular transport and signal transduction, and are also prominent in neurodegenerative pathology. Protein folding to attain minimal free energy is assisted by a variety of chaperone proteins. Any aberration in the protein folding process may lead to accumulation of unfolded/misfolded proteins, resulting in “Endoplasmic Reticulum stress” (ER stress).

Like most proteins, IDPs are susceptible to multiple PTMs; in neurological diseases, excessive PTMs may alter protein structure, favor binding to novel partners, and promote aggregation. Neurons have extensive protein-repair capacity that helps them to detect and salvage misfolded proteins, thereby preventing or reducing ER stress and thus ameliorating neurological damage (Jellinger, 2010; Cristofani et al., 2020). The accumulation of misfolded or intrinsically disordered proteins is recognized as a common characteristic of many neurodegenerative diseases (Uversky, 2015; Ayyadevara et al., 2021). IDPs have unique plasticity, conformational adaptability, and ability to bind to multiple partners — conferred by diverse properties such as structural malleability, low hydrophobicity, high solvent-accessible surface area, and high abundance of charged and polar residues (Wright and Dyson, 2015; Salvi et al., 2019).

Misfolded proteins are also known to cause cytotoxicity through toxic gain of function (Soto and Pritzkow, 2018). Many misfolded proteins/peptides such as Aβ_1–42_, hyper-phosphorylated tau (hP-tau), α-synuclein, and others, are involved in synaptic signaling pathways (Ashraf et al., 2014). For example, α-synuclein is a presynaptic protein that can relocate to mitochondria where it disrupts protein import; whereas mutated or hyperphosphorylated tau disrupts microtubule function (Stefanis, 2012; Melo et al., 2018). Liquid-liquid phase separation is an early event in the formation of aggregates featuring key neuropathology-associated proteins such as α-synuclein in Parkinson’s disease (Ray et al., 2020). Microtubule-associated protein tau normally stabilizes neuronal microtubules, but over time, and especially when hyperphosphorylated, tau can undergo liquid-liquid phase separation leading to microtubule nucleation and irreversible aggregation (Wheeler, 2020). Numerous misfolded proteins appear among the constituents of aggregates associated with Alzheimer’s disease (AD) (Ayyadevara et al., 2016a). Although specific aggregate components distinguish among different neurological diseases, such as AD, Parkinson’s disease (PD), and Amyotrophic Lateral Sclerosis (ALS), these pathologies all involve similar processes of protein misfolding and aggregate accrual. Although disease-associated aggregate proteins exhibit considerable diversity in sequence, size, structure, and function, after misfolding most form intermolecular β-sheet-rich structures ranging from small oligomers to large aggregates. Since not all disordered proteins end up in aggregates, we sought to identify properties that distinguish disordered proteins that are destined for aggregation, from those that are not associated with neuropathology.

## Materials and methods

### Selection of proteins from DisProt database

The DisProt database^1^ is a manually curated database of intrinsically disordered proteins. DisProt has been updated over the last 14 years, including addition of attributes such as structural/functional aspects of protein domains. DisProt has its own set of descriptors for each protein, including state(s), state transitions, and “disorder ontology.” Twenty three disordered proteins were selected from DisProt to test the effects of their knockdowns on aggregation and aggregation-dependent traits in *C. elegans* models of neuropathogenic aggregation.

### *Caenorhabditis elegans* strains

All *C. elegans* strains were grown under standard conditions at 20°C unless otherwise noted. Four transgenic strains were used in this study. (***i.***) **CL2355** [pCL45 (*snb-1:Aβ_1–42_: 3’ UTR* (long) + *mtl-2:gfp*], a strain with pan-neuronal expression of a human Aβ_1–42_ transgene, causing deficits in chemotaxis, associative learning, and thrashing in liquid media. (***ii.***) **CL4176** [*dvIs27; myo-3p:Aβ_1–42_:let-851 3’ UTR*) + *rol-6(su1006)*], a strain with muscle expression of human Aβ_1–42_. CL2355 and CL4176 produce low levels of Aβ_1–42_ at 20°C but progress to chemotaxis or paralysis, respectively, with age or after upshift to 25.5°C (Balasubramaniam et al., 2019; Ayyadevara et al., 2021). (***iii.***) **NL5901** [*unc-54p:α-synuclein:yfp* + *unc-119(*+)] expresses YFP-tagged human *α-*synuclein in muscle, resulting in progressive paralysis. (***iv.***) **AM141** [*unc-54*/*q40:yfp*] expresses Q40:YFP in muscle, leading to adult accumulation of YFP-fluorescent foci and late paralysis. All strains were obtained from the Caenorhabditis Genetics Center. *Escherichia coli* strain OP50 was replaced as the bacterial food source for RNAi exposure by *E. coli* HT115, harboring a vector that expresses RNAi constructs as dsRNA; these substrains were selected from the Ahringer RNAi Library (Kamath and Ahringer, 2003).

### RNA interference

Selected genes, encoding IDPs listed in DisProt that were also implicated by proteomics showing enrichment in both Parkinson’s and Alzheimer’s aggregates (Balasubramaniam et al., 2019), were subjected to RNAi-mediated knockdown by feeding them target-specific RNAi bacteria from the Ahringer library (Kamath and Ahringer, 2003). Synchronously harvested eggs were transferred to plates seeded with selected sublines of *E. coli* strain HT115 (DE3) that transcribe double-stranded RNA corresponding to an exonic segment of each targeted gene, cloned into the L4440 plasmid multiple-cloning site (Kamath and Ahringer, 2003). Control worms were fed bacteria carrying L4440 without an exonic insert (“feeding vector” or “FV” controls).

### Paralysis assay

Synchronous cohorts of the CL4176 strain, expressing Aβ_1–42_ in body-wall muscle, were initiated by lysing adult worms in alkaline sodium hypochlorite solution (Ayyadevara et al., 2001, 2008). Unlaid eggs recovered from lysed worms were transferred onto 60-mm agar plates seeded with bacteria expressing dsRNAs against targeted genes (see preceding section). Worms in all groups were upshifted from 20° to 25.5°C at the L3/L4 transition (47–49 h after lysis of parental worms) to induce expression of Aβ_1–42_ (Ayyadevara et al., 2016a). Paralysis of worms (defined as loss of touch-response motility) was scored at 19, 27, and 42 h post-upshift, until the longest-surviving group exceeded 50% mortality. To slow development of progeny in synchronized populations, 5-fluoro-2’-deoxyuridine (FUdR) was added to RNAi plates and control (FV) plates, at a final concentration of 2 μM, each containing worms from pre-gravid (L4/adult molt, day 2.5 post-hatch) through post-gravid ages (beyond 6–7 days post-hatch).

### Fluorescence imaging of polyglutamine and alpha-synuclein aggregates

Aggregates in strain AM141, expressing Q40:YFP in muscle which forms punctate aggregates in adult worms, were analyzed for number and intensity of aggregates using FIJI (ImageJ2) (Schneider et al., 2012). Parameters and exposure were kept constant for each experiment to avoid bias. To visualize GFP aggregates, worms were collected and immobilized using sodium azide, and their images captured at 10× magnification using a Keyance fluorescence microscope. Counts of Q40:YFP aggregates per worm, and total punctate fluorescence per worm, were calculated for 4–8 worms per field, and 5–6 fields per group.

Alpha synuclein inclusions in body-wall muscle of strain NL5901 were analyzed for the average intensity of YFP expression, using FIJI (ImageJ2) while maintaining uniform conditions. Very similar results were obtained with strain OW13 (a genetically identical construct created independently; data not shown). Each experiment was initiated with synchronized eggs from well-fed worms, lysed with alkaline hypochlorite; eggs were transferred to NGM plates seeded with OP50 for maintenance, or HT115 for knockdown experiments. RNAi KD worms were grown on different RNAi-expressing HT115 clones, at 20°C. Images of worms were captured at adult days 1 and 5 (24 h or 5 days after the L4/adult molt) to quantify α-synuclein aggregation based on punctate YFP signal.

### Data collection and descriptor retrieval

Parkinson’s disease (PD) aggregates were processed to separate sarkosyl-soluble from sarkosyl-insoluble aggregates. Recovered aggregates were digested with trypsin for proteomic analyses by LC-MS/MS as previously described (Ayyadevara et al., 2016b). Mass spectrometry data were collected, along with protein-sequence-based analyses, to compile descriptors (Table 3), and to calculate physiochemical properties and disorder scores, etc. using PONDR^2^, ESpritz Version 1.3^3^, and Aggrescan^4^. PONDR derives 5 predictions from the FASTA sequence of each protein: VLXT, XL1_XT, CAN_XT, VL3-BA, and VLS2 (Xue et al., 2010). ESpritz produces protein disorder scores based on a choice of prediction tools (e.g., X-Ray, NMR, and Disprot) (Walsh et al., 2012), from which we selected NMR. Both PONDR and ESpritz were set to thresholds of 40% to predict disorder, but numerical output values were used as inputs to NN and SVM. These packages were augmented with Python code to calculate hydrophobicity, aromaticity, percent of individual amino acids, percent basic and acidic amino acids, etc. PSPredictor^5^, a second-generation, sequence-based tool to predict the potential of each protein for liquid-liquid phase-separation (Chu et al., 2022), was set to ≥ 0.5 threshold; actual numerical output values were used as inputs to NN and SVM.

### Neural network, support vector machine, and principal component analyses

Algorithms for Neural Network (NN), Support Vector Machine (SVM), and Principal Component Analysis (PCA) were implemented, trained and tested using Orange™ software (Demsar et al., 2013) to generate and visualize the outputs.

For Neural Networks, Orange™ employs a multilayer perceptron algorithm with back-propagation, splitting the dataset randomly 80:20 into training and testing sets. NN was assessed with a range of input parameters (Supplementary Table 1). The configuration with highest AUC had 300 hidden layers and 1350 iterations; activation method was set to “ReLu,” solver selected as “SGD” and numerical tolerance was set to 0.0005 (Deng et al., 2015).

Support Vector Machine (SVM) is a machine-learning method used for classification, regression and outlier detection, in which linear regression is performed in a high-dimension feature space. Where possible, SVM imputes missing values as means of existing values; otherwise, SVM removes instances with unknown target values and empty columns. SVM was assessed with a variety of input parameters (Supplementary Table 2), selecting those producing the maximal AUC (cost = 0.30, regression loss = 0.40, kernel = 0.01, numerical tolerance = 0.00011, iterations = 100).

Principal component analysis (PCA) is a stepwise, forward/reverse multivariate linear regression performed within Orange™ (Demsar et al., 2013) to identify orthogonal clusters of input parameters. These clusters collapse highly correlated predictors into a minimal set of uncorrelated (“orthogonal”) predictor dimensions computed from network graphs for the nodes of interest (Jolliffe and Cadima, 2016). PCA applies linear transformations to fit all 49 predictors into a coordinate system in which the most significant variance component is represented by the first component (PC1), and each successive component is orthogonal to all others and accounts for a smaller fraction (%) of total variance (Giuseppe et al., 2013). The first six PCs accounted for >88% of total variance.

### Structural dynamics of disordered proteins

Simulations were performed using the GROMACS simulation package implemented via the WebGRO server^6^ developed in-house. Each target protein was immersed in a triclinic box containing simple point charge (SPC) water. GROMOS96 43A force field was added evenly to the simulation system. The simulation system was neutralized by adding NaCl as counterions, and NaCl was supplemented to 0.15 M to approximate the physiological salt concentration. The whole system was energy minimized using Steepest Descent method for 5000 steps, and then equilibrated using the NVT/NPT method for 300 picoseconds. Each MD run used the leap-frog integrator for 200 ns; simulation trajectories were identified with the GROMACS trajectory analysis package and plotted using XMGRACE.

### Progeny production assay

Equal numbers of synchronized L1 worms (strain AM141, expressing *unc54/q40:yfp* in muscle) were placed on 100-mm agar plates and maintained at 20°C. Worms matured into gravid adults in 2.5 days, and the number of progeny produced during days 5 and 6 post-hatch were counted for triplicate plates, calculating the average number of progeny per plate per day. Significance was determined by a 2-tailed heteroscedastic *t*-test.

## Results

### Understanding the physiological effects of known disordered proteins in *Caenorhabditis elegans* neurodegenerative-disease models

#### A nematode model of neuronal amyloidosis

**FIGURE 1:**
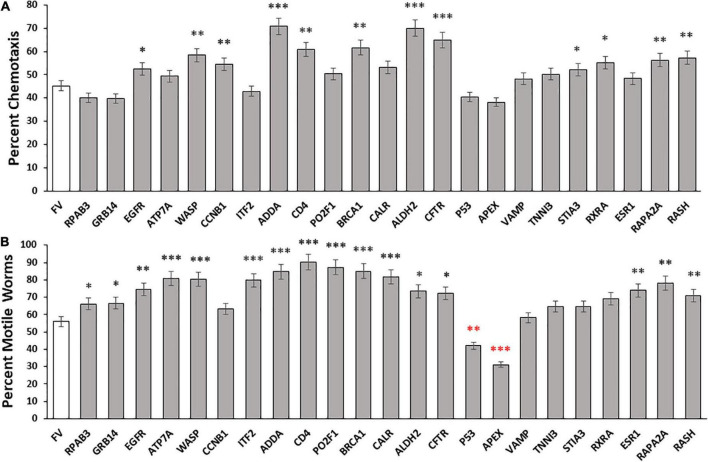
Aggregate abatement by knockdown of intrinsically disordered proteins (IDPs) from Disprot. **(A)** Histogram showing means ± SEM for calculated average chemotaxis of *Caenorhabditis elegans* strain CL2355 [*Aβ_1–42_:3’UTR(long*)], a model featuring pan-neuronal expression of human Aβ_1–42_ peptide, which leads to a 50 – 60% deficit in chemotaxis relative to young-adult wild-type worms (*N* = 100–150 worms per group). **(B)** Histogram show mean ± SEM for paralysis of *C. elegans* strain CL4176, an induced-Aβ_1–42_ model of AD-like amyloid deposition after muscle expression of human Aβ_1–42_ (*N* = 200–250 worms per group). Significances of differences from controls by heteroscedastic, 2-tailed *t* tests are *P ≤ 0.05; ^**^P ≤ 0.01; ^***^P ≤ 0.0001; ^****^P ≤ 0.00001. Red asterisks indicate values significantly *below* controls.

To investigate the roles of disordered proteins in aggregation, we assessed 23 high-confidence IDPs from DisProt, a manually curated IDP database. The selected IDPs were tested in *C. elegans* strain CL2355, after induction of human Aβ_1–42_ peptide synthesis in all neurons. RNAi-mediated knockdown of each gene was initiated at hatch, and day-5 post-hatch worms were assayed for chemotaxis toward n-butanol, a behavior that deteriorates gradually with age and acutely upon induced neuronal expression of the Aβ_1–42_ peptide (Balasubramaniam et al., 2019). Of 23 tested IDP knockdowns, 12 (53%) conferred significant protection against loss of chemotaxis following Aβ aggregate formation, relative to controls, whereas 5 knockdowns had no effect or may have slightly worsened the trait (Figure 1A). For the 12 protective knockdowns, chemotaxis rescued 15–51% of the deficit attributed to neuronal Aβ_1–42_ expression, implying that each protein contributes to neuronal Aβ_1–42_ aggregation and the associated loss of chemotaxis. It is of particular interest that knockdown of 11 other genes encoding disordered proteins (*RPAB3, GRB14, ATP7A, ITF2, PO2F1, CALR, P53*, APEX, *ESR1*, *VAMP*, and *TNNI3*) did not significantly improve chemotaxis, suggesting that their encoded proteins contribute little to chemosensory deficit caused by Aβ_1–42_ aggregation, or are functionally redundant with other genes or pathways.

#### A nematode model of muscle amyloidosis

We next evaluated the roles of these disordered proteins in a *C. elegans* model of muscle Aβ_1–42_ aggregation. We knocked down expression of the same disordered proteins in *C*. *elegans* strain CL4176, which expresses Aβ_1–42_ peptide in muscle, leading to Aβ_1–42_ aggregation and ensuing paralysis. The fraction of paralyzed worms was assayed in day-4.5 adults (i.e., 47 hr. after the L4/adult molt; Figure 1B). Sixteen of 23 knockdowns (70%) elicited significant rescue, restoring 23 – 78% of the motility lost due to Aβ_1–42_-mediated aggregation in muscle. However, five knockdowns did not cause a significant shift in paralysis, and two (*P53* and *APEX*) significantly *decreased* motility (red asterisks in Figure 1B).

Although the two assays evaluated disparate effects of Aβ_1–42_ aggregation in different tissues, the Pearson correlation between these results is significant (*R*_*P*_ = 0.59; *P* < 0.0025). We recognize that the efficiency of RNAi knockdown may be reduced for some neuronal genes. The 23 IDPs listed in the paper include 18 with documented neuronal expression (see WormBase.org), of which 16 (89%) were successfully suppressed by RNAi, whereas for the 5 gene targets with no detectable neuronal expression, only one (20%) impacted the chemotaxis phenotype upon knockdown. As summarized in Table 1, RNAi knockdown of neuronally expressed genes was nearly 6-fold more effective in disrupting a behavioral trait (chemotaxis) than was KD of neuronally silent genes (*P* < 0.004). In contrast, there was a 2.4-fold change in KD efficacy (not significant) for paralysis, a trait mediated by genes expressed in both muscle cells and neurons (Table 1). Together, these data imply that disordered proteins play crucial roles in aggregate formation across diverse neurodegenerative-disease models, impacting motility and chemosensory behavior, along with other physiological processes.

**TABLE 1:** RNAi knockdowns have far greater effects on a neuronally-mediated trait (chemotaxis) for KD targets with documented neuronal expression.

	*N*	Chemotaxis		Paralysis	
		Mean effect	SEM	Mean effect	SEM
KD of genes with documented neuronal expression	18	17.8%	2.4%	26%	3.0%
KD of genes lacking neuronal expression	5	3.0%	3.0%	11%	5.9%
Fold difference		5.9		2.4	
Significance (2-tailed *t* test)		0.0034		0.056 (N.S.)	

### Proteins in Parkinson’s aggregates are enriched for intrinsically disordered proteins (IDPs)

We observed a substantial number of intrinsically disordered proteins in AD hippocampal aggregates (Ayyadevara et al., 2016a), many of which had also been previously identified in aggregates isolated from other neurodegenerative diseases (Vaquer-Alicea and Diamond, 2019) and also found in aging human skeletal muscle, and hearts and brains from aged or AD-model mice (Ayyadevara et al., 2016b,^2019^; Kakraba et al., 2019). These commonalities suggested that similar processes may be involved in a diverse array of age-associated pathologies, possibly involving a conserved set of IDPs. To pursue that possibility, we asked whether PD brain aggregates are also enriched for disordered proteins. We predicted the disordered fraction of each protein identified by proteomic analysis of Parkinson’s disease tissue, in both soluble and detergent-insoluble aggregates, using the PONDR and ESpritz on-line servers (Xue et al., 2010; Walsh et al., 2012). Results were similar; only PONDR outputs are shown here.

These servers predicted that 53% of the proteins identified in Parkinson’s aggregates have at least 40% disorder, an enrichment >1.7-fold above the 31–32% predicted for human proteins overall (Ali and Ivarsson, 2018; Deiana et al., 2019) (Chi-squared *P* < 10^–4^). Of 845 IDP proteins enriched in insoluble aggregates from PD, 632 (75%) were also significantly enriched in AD aggregates (each relative to similar aggregates from age-matched controls) (Ayyadevara et al., 2016a). A subset of 197 aggregate-enriched proteins shared by AD and PD, selected for a broad range of disorder scores (Table 2), indicates very little correlation between AGGRESCAN-predicted aggregation propensity and estimated disorder (*R*_*P*_ = 0.095).

**TABLE 2:** Selected proteins shared by Alzheimer’s disease (AD) and Parkinson’s disease (PD) aggregates, with a range of disorder scores.

Protein name	Aggregation propensity	Percent disorder
LPPRC_HUMAN	137.2	68.4
KIF1A_HUMAN	125.6	44.3
RPN1_HUMAN	84.3	50.3
E41L3_HUMAN	69.6	72.4
MPP6_HUMAN	42.2	100
SYN1_HUMAN	33.5	49.8
CLUS_HUMAN	28.6	59.6
STX1A_HUMAN	27.3	74.3
SYNEM_HUMAN	26.8	46.7
1433F_HUMAN	21.5	45.9
1433Z_HUMAN	21.0	53.8
1433B_HUMAN	20.3	54.4
1433G_HUMAN	20.1	46.1
APOE_HUMAN	15.5	74.7
GFAP_HUMAN	9.8	82.4
SNX3_HUMAN	3.0	13.0
STMN1_HUMAN	2.6	25.3
HSPB1_HUMAN	2.9	15.0
NEUM_HUMAN	1.2	35.9
MARCS_HUMAN	1.8	40.5
H10_HUMAN	5.7	97.6
K1C9_HUMAN	4.2	66.6
BASP1_HUMAN	0	100

Aggregation propensity was predicted by AGGRESCAN; disorder scores were generated by PONDR.

The Pearson correlation coefficient between these two parameters was 0.095 (not significant).

**TABLE 3:** Descriptors in top 6 PCs, accounting for 88% of dataset variance.

	Criteria for inclusion in insoluble aggregates	Criteria for aggregate exclusion
PC1	PONDR disorder score (>0.607) (Deng et al., 2015)	PONDR disorder score (<0.607)
PC2	α-synuclein insoluble-aggregate content (spectral hits > 8)	α-synuclein soluble-aggregate content (spectral hits < 42)
PC3	Percent amphipathic residues (>5%)	Percent acidic residues (>6.7)
PC3	Number of disordered regions > 30 amino acids (>4)	Total hot-spot area of aggregation (<0.017)
PC4	AGGRESCAN aggregation propensity per 100 a.a. (Kikis et al., 2010) (>3.2)	
PC5	Percent aromatic residues (≤21.6%)	
PC6	Percent basic residues (6.7 < %basic ≤ 10.2)	Number of disordered segments (<3)
PC6		Number of disordered residues (<53)

Each successive Principal Component accounts for the maximal fraction of remaining variance. The threshold criterion for classification into “insoluble aggregates” is shown in parentheses.

### Machine-learning/neural-network descriptors predict aggregation

The above data on IDPs led us to re-evaluate the relationship between the disorder level and other properties of a protein, and its tendency to enter into aggregates. To understand which physiochemical properties might favor aggregation of disordered proteins, we selected 400 disordered proteins enriched in both PD and AD aggregates, and compiled the numbers of spectral hits for each such protein in the detergent-soluble and -insoluble fractions. The resulting dataset also listed 49 distinct physiochemical properties for each protein, including disorder score, hydrophobicity, aggregation score, aromaticity, and percentage of several other key amino acids. Machine-learning software then randomly partitioned the list 80:20, into sets used for training and testing respectively; this process (partitioning, training and testing) was repeated for 50 permutations. We then categorized the proteins based on their PD-aggregate spectral hits into four groups: “INSOL,” where the protein is only found in insoluble aggregates; “SOL,” where the protein is found only in soluble aggregates; “BOTH,” where the protein is found in both soluble and insoluble aggregates; and “NOAGG” for proteins not found in any aggregates.

We trained Principal Component Analysis (PCA), neural-network (NN), and support-vector-machine (SVM) algorithms to predict a protein’s probability of entering aggregates. The neural-network and SVM predictions resulted in 79.5 and 80% accuracy, respectively, for the testing groups — indicating equivalent performance. This suggests that, among 49 input descriptors, there may be a subset of properties that determine aggregation propensity of disordered proteins, supporting our hypothesis that disorder alone does not dictate aggregation. The PCA, NN, and SVM algorithms used physiochemical properties, including net hydrophobicity, % acidic residues, % basic residues, % charged and uncharged residues, total aggregation-prone expanse, total hot-spot aggregation expanse, total number of disordered segments, longest disordered region, and overall% disorder, augmented by *in silico* predictors of protein disorder (Table 3).

**FIGURE 2:**
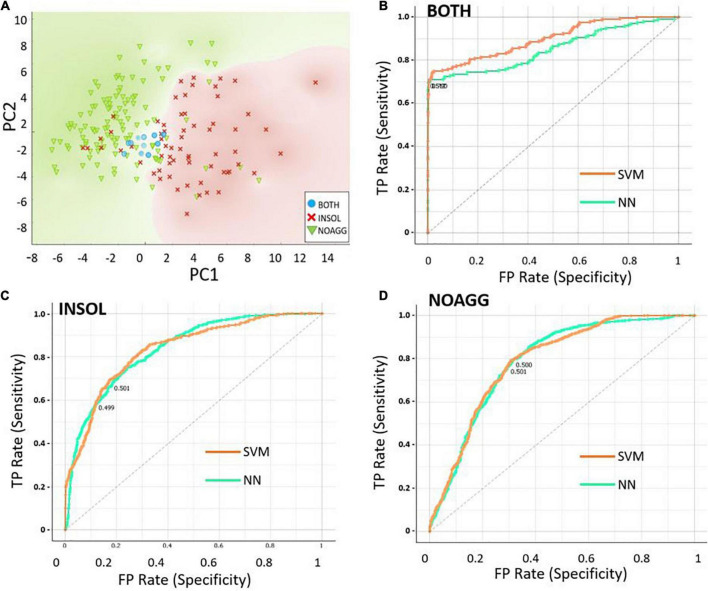
Data partitioning based on the first two principal component analysis (PCA) components. **(A)** Scatter-plot illustrating 2-dimensional sorting of intrinsically disordered proteins (IDPs) based on the first two components, PC1 and PC2, into categories INSOL (sarkosyl-insoluble aggregates), NOAGG (not aggregated), and BOTH (included in both soluble and insoluble aggregates). **(B–D)** Receiver-Operating Characteristic (ROC) analysis of the 3 classes, INSOL, NOAGG, and BOTH, showing similar curves for NN and SVM, for total positives (TP, y axis) *vs*. false positives (FP, x axis) — representing sensitivity and specificity, respectively.

To identify a minimal set of descriptors or properties required for prediction accuracy, we used Principal Component Analysis (PCA) to compare predictive models. Disorder scores generated by the PONDR program, which itself uses neural networks to identify disordered protein regions, was the most influential predictor of aggregate inclusion (PC1 in Table 3), followed by spectral counts in α-synuclein insoluble aggregates (PC2). The first 6 principal components accounted for > 88% of dataset variance. Limiting descriptor inputs to the top 3 PCA components reduced SVM and NN accuracy (AUC) by a further 4.6% (see Table 4). A 2-dimensional partitioning of proteins by aggregation propensity, based on the first two components, is illustrated in Figure 2A. Predictions using both PC1 and PC2 show a correlation coefficient *R* of 0.895, and *R*^2^ = 0.80, with actual detection in PD and AD aggregates. Receiver-Operating Characteristic (ROC) analyses, plotting sensitivity vs. specificity, are displayed for neural network and support vector machine predictions for each of the classes BOTH, INSOL, and NOAGG (Figures 2B–D, respectively). The SOL category was poorly resolved from other classes (Supplementary Table 3). We also predicted potential liquid-liquid phase separation for each protein in the dataset using PSPredictor. PSPredictor scores correlated fairly well with NN and SVM predictions, producing linear (Pearson) correlation coefficients of 0.75 and 0.85 with NN and SVM predictions, respectively. Spearman rank-order correlations were slightly higher at 0.81 for NN and 0.90 for SVM predictions (Supplementary Table 4, last line).

**TABLE 4:** Accuracy of machine-learning algorithms vs. number of PC inputs used.

Machine learning model	AUC (top 6 PCs)	AUC (top 5 PCs)	AUC (top 4 PCs)	AUC (top 3 PCs)
Support vector machine (SVM)	0.826	0.798	0.795	0.789
Neural network	0.817	0.789	0.784	0.781

AUC is determined within Orange™ for all ROC results from 50 data permutations (Demsar et al., 2013).

### Structural analysis of implicated disordered proteins

Since the majority of disordered proteins are predicted to be structurally unstable, and are thus likely to unfold more readily than other proteins (Uversky, 2019), we analyzed the structural dynamics of several IDPs that were enriched in both PD and AD insoluble aggregates. We selected tubulin beta-4A chain (TUBB4A), a disordered monomer that polymerizes into the highly ordered and stable microtubule structure; glial fibrillary acidic protein (GFAP), an intermediate filament protein with a disorder score of ∼86%; RAP2A, a small GTP-binding protein related to Ras, which forms a signaling complex with NEDD4 and TNIK regulating neuronal dendrite morphogenesis; and three 14-3-3 paralogs (α, γ, and σ), members of a small family of conserved signaling molecules responsive to protein phosphorylation. Each of these aggregate-enriched proteins was consistently abundant (50–485 spectral counts) in each of the immunopurified aggregate types (sarkosyl-insoluble aggregates isolated by antibody affinity for α-synuclein, Aβ_1–42_, or tau), with the exception of RAP2A, which was substantially less abundant (25 total hits). Spectral counts for individual proteins in each aggregate class were roughly in proportion to total sarkosyl-insoluble aggregate protein (see percentages in Table 5).

**TABLE 5:** Spectral hits for proteins in Parkinson’s disease (PD) brain aggregates.

Protein	α-synuclein aggregates	Aβ_1–42_ aggregates	tau aggregates	Total
GFAP	755 (48.1%)	428 (27.3%)	386 (24.6%)	1569
TUBB4A	485 (57.7%)	194 (23.1%)	161 (19.2%)	840
RAP2A	18 (72%)	4 (16%)	3 (12%)	25
14-3-3S	135 (55.8%)	57 (23.6%)	50 (20.7%)	242
14-3-3G	116 (49.6%)	58 (24.8%)	60 (25.6%)	234
14-3-3Z	164 (54.3%)	71 (23.5%)	67 (22.2%)	302
Sark-insol. aggregates	58,813 (55.3%)	27,924 (26.3%)	19,534 (18.4%)	106,271
Sark-sol. aggregates	18,997 (35.7%)	15,984 (30.1%)	18,176 (34.2%)	53,157

Protein spectral counts are totals for each immunopurified, sarkosyl-insoluble aggregate type.

**FIGURE 3:**
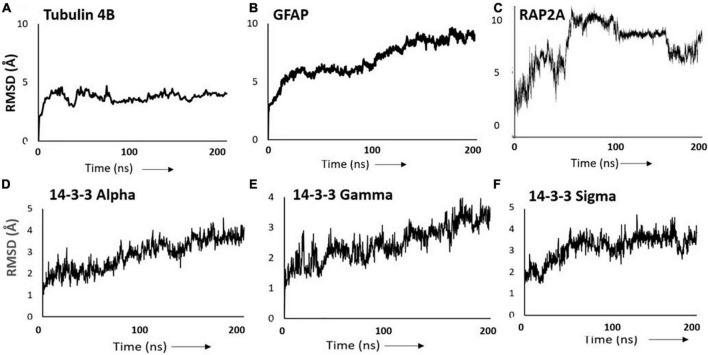
RMSD plots of disordered proteins. Intrinsically disordered protein (IDP) structures were simulated for 200 ns: **(A)** Tubulin 4B; **(B)** glial fibrillary acidic protein (GFAP); **(C)** RAP2A; **(D)** 14-3-3α (alpha); **(E)** 14-3-3γ (gamma); **(F)** 14-3-3σ (sigma). RMSD instability reflects random structural perturbations over time.

We predicted structural dynamics of these six proteins using atomistic molecular-dynamic simulations of the monomeric forms, conducted for 200 ns in triplicate (Figure 3). All six proteins are predicted to show RMSD instability, based on average tracings of three 200-ns simulations for each protein. Tubulin β chain 4B is the most stable of these, but nevertheless undergoes RMSD fluctuations of 10–20% for at least 200 ns (Figure 3A). The RMSD of GFAP continued to expand throughout the 200-ns simulations, indicative of progressive unfolding (Figure 3B). RAP2A showed RMSD fluctuations of > 50%, and beyond ∼70 ns it appeared to oscillate between two or more metastable conformations (Figure 3C). The three 14-3-3 paralogs (Figures 3D–F) were predicted to expand progressively over the course of the simulations.

### Intrinsically disordered proteins (IDPs) in Parkinson’s disease (PD) aggregates influence stress survival and reproduction

We previously reported that RNAi knockdowns that suppress expression of orthologs of AD aggregate-enriched proteins conferred significant protection from pathology-associated outcomes in *C. elegans* models of neurodegenerative aggregation (Ayyadevara et al., 2015, 2016a,^2017^, ^2021^). To assess whether knockdowns are similarly protective for target IDPs implicated by our SVM and NN algorithms, we quantified aggregate formation and progeny production after RNAi-mediated knockdown of *C. elegans* orthologs of six representative IDP genes with ≥ 40% disorder, enriched in both AD and PD aggregates (*DHX9, PLEC, FABPH, TUBB4, GFAP*, and *MPPA*).

#### Influential intrinsically disordered protein (IDPs)’s tested in nematode models of neuropathic aggregation

**FIGURE 4:**
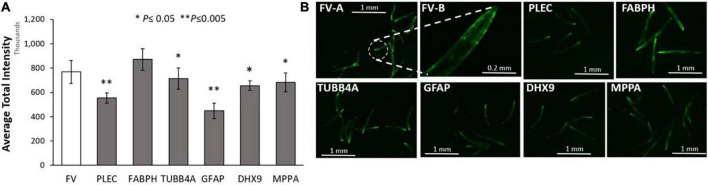
Aggregation of human α-synuclein after KD of Alzheimer’s disease (AD)/ Parkinson’s disease (PD)-aggregate Intrinsically disordered proteins (IDPs). **(A)** Histograms for worms exposed to RNAis of IDPs at 20°C, imaged as day-5 post-hatch adults. Six IDP knockdowns were exposed continuously from hatch, to RNAi targeting IDPs shared by AD and PD aggregates. **(B)** Images are shown of *Caenorhabditis elegans* day-5 adults of strain NL5901 [*unc-54p:alpha-synuclein:YFP* + *unc-119(*+)], a model of PD-like α-synuclein aggregation, with YFP fluorescent foci appearing in body-wall muscle. Experimental groups differ from controls with significance based on heteroscedastic, 1-tailed *t* tests: *P ≤ 0.05; ^**^P ≤ 0.005. FV-A and other images are shown at 4× magnification; FV-B is shown at 20× magnification.

We first assessed the effects of knockdowns targeting orthologs of these IDPs in a *C*. *elegans* model of α-synuclein aggregation, characteristic of PD. To visually monitor the consequences of each knockdown, we employed *C*. *elegans* strain NL5901, expressing α-synuclein fused to yellow fluorescent protein [*unc-54p:alpha-synuclein:yfp* + *unc-119* (+)] in body-wall muscle. We quantified YFP inclusions in muscle of control worms, vs. worms subjected to RNAi knockdowns targeting *C. elegans* orthologs of the 6 IDP genes that encode proteins enriched in AD and PD aggregates. RNAi exposure extended from hatch until aggregate assessment 5 days later. Based on mean YFP intensity per worm, these IDP knockdowns decreased α-synuclein aggregate load by 15–43% (Figure 4), with *GFAP* KD exerting the greatest effect, followed by *PLEC*.

**FIGURE 5:**
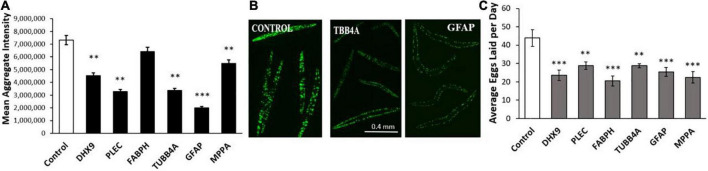
Polyglutamine aggregation and fertility after KD of Alzheimer’s disease (AD)/Parkinson’s disease (PD)-aggregate IDPs. **(A)** Average aggregate fluorescence per worm in *Caenorhabditis elegans* strain AM141 (expressing *q40:yfp* in body-wall muscle), a model of HD huntingtin-like aggregation. Six IDPs were assessed following exposure to RNAi for the indicated IDPs, shared by both AD and PD. **(B)** Images are shown (10× magnification) of *C. elegans* AM141 adults, at 5 days post-hatch after 4 days exposure to RNAi targeting β-tubulin or GFAP. **(C)** Average number of eggs laid during days 5 and 6 post-hatch following knockdown with the indicated RNAi. Differences from controls are significant by heteroscedastic, 1-tailed *t* tests at *P ≤ 0.05; ^**^P ≤ 0.01; ^***^P ≤ 0.0001.

We next assessed these same six IDPs in a *C*. *elegans* model of age-progressive, huntingtin-like aggregate formation (distinct fluorescent foci arising from YFP-tagged Q40 expressed in muscle). RNAi suppression of these IDPs decreased Q40:YFP punctate fluorescence per worm by 12–73% (Figures 5A,B). Both the number and intensity of aggregates were reduced by suppression of these IDPs, with *GFAP* and *PLEC* again exerting the greatest effect, followed closely by *TUBB4A* (tubulin β chain 4A) (Figure 5A). We noticed fewer progeny issuing from these KD groups, leading us to quantify fecundity (Figure 5C). Intriguingly, each IDP knockdown significantly lowered the average number of eggs laid on days 5 and 6 post-hatch, by 40–60% relative to progeny of feeding-vector control worms (*P* ≤ 0.001 to *P* ≤ 0.0001). This observation implies that each IDP contributes to fertility and/or development; that is, each gene product serves a positive function early in life, although most become deleterious subsequently.

## Discussion

By far the most influential descriptor, for prediction of aggregate inclusion, was the PONDR disorder score (PC1 in Table 3). This confirms that disorder is the predominant feature determining protein accretion into aggregates – which has been widely assumed and was strongly supported by our observation of highly significant IDP enrichment in protein aggregates. PC2 is simply the total insoluble-aggregate content of α-synuclein, which Table 5 reveals to be a reasonably good surrogate for the sarkosyl-insoluble protein content of any aggregate class. This supports our hypothesis that common processes of accrual mediate the formation of all aggregate varieties. Additional increments, although of ever-diminishing importance, are provided by PC3–PC6. It is noteworthy that all PCA dimensions are deemed to be independent of one another, meaning that there is no discernable correlation between disorder score (PC1), insoluble aggregate burden (PC2), the number of extensive disordered regions (PC3) or the% amphipathic, acidic, aromatic, or basic residues (PC3, PC5, PC6). AGGRESCAN provides a sequence-based prediction of aggregation propensity, which appears as PC4. At this level, we cannot be certain that this descriptor is truly orthogonal to all other input variables, but it is clear that AGGRESCAN provides less valuable information than PONDR (PC1) or aggregate burden (PC2).

Protein folding is obligatory for generation of functional proteins (Diaz-Villanueva et al., 2015). Most newly synthesized proteins will reach a native conformation upon completion of synthesis; proteins that are initially misfolded are assisted by chaperones to assume stable conformations (Hwang and Qi, 2018). Multiple mechanisms provide redundancy, and help to minimize the loss of functionally robust structures (Hwang and Qi, 2018); nevertheless, it is estimated that over half of newly synthesized proteins may be degraded co-translationally (Turner and Varshavsky, 2000). IDPs have been reported to be involved as key regulators of diverse and essential physiological processes including transcription, translation and cell signal transduction (Wright and Dyson, 2015). In a recent study, Cuevas-Velazques et al. demonstrated that IDPs coupled to fluorescent tags can serve as biosensors of osmotic stress (Cuevas-Velazquez et al., 2021). We here add the novel observation that IDPs contribute to reproductive potential (Figure 5C).

Multiple proteins, and especially IDPs, coalesce into aggregates, which also contain specific RNA and DNA sequences to which many IDPs bind (Shmookler Reis et al., 2021). Disease-associated aggregate components serve as diagnostic biomarkers for diverse neurodegenerative pathologies (Balasubramaniam et al., 2019; Ayyadevara et al., 2021). To evaluate the importance of these IDPs for aggregate formation and associated traits, we performed an initial screen in which IDPs from the DisProt database were individually knocked down in *C. elegans* by RNA interference. Most KDs conferred substantial protection against aggregate formation, and/or protected against age-progressive traits used as end-points in *C. elegans* models of diverse neurodegenerative diseases. We observed similar protection in studies of the human SERF2 protein (Balasubramaniam et al., 2018) and its *C. elegans* ortholog CRAM-1 (Ayyadevara et al., 2015). Each IDP KD conferred similar protection against aggregate accrual, a hallmark feature of aging and age-associated diseases (van Ham et al., 2010).

Numerous IDPs play key functional roles during high energy-demand states such as reproduction and response to stresses encountered during development. Nevertheless, the age-progressive increase in protein aggregation, which is largely post-reproductive, will be exacerbated by the tendency of these sticky proteins to interact non-randomly with other protein partners, or with RNA or DNA (Marcelo et al., 2021; Shmookler Reis et al., 2021), and in response to stressors in the cell environment (Balasubramaniam et al., 2019).

Individual IDPs may qualify as instances of “antagonistic pleiotropy” (Williams, 1957), wherein allele-specific survival and/or reproductive value drive natural selection early in life, unhindered by detrimental effects arising later. We found that 6 out of 6 IDP knockdowns reduce *C. elegans* reproductive fitness, evidenced by reduced fecundity (Figure 5C), despite decreasing protein aggregation (Figure 5A) and its deleterious sequelae that reduce long-term survival (Ayyadevara et al., 2016a,b). Antagonistic pleiotropy is not an obligatory property of natural gene variants, but is observed for a subset of longevity-associated alleles (Ayyadevara et al., 2001). We expect IDPs to play important roles in reproduction, perhaps due to their ability to bind multiple partners and thus coordinate multiple pathways. With aging, however, IDPs may become increasingly sensitive to progressive changes arising from oxidation and inflammation, ultimately impairing proteostasis (Kikis et al., 2010; Bektas et al., 2018). Non-random interactions of these IDPs with protein and nucleic-acid partners may contribute to aggregate initiation and progression (Stefanis, 2012; Ayyadevara et al., 2017, 2021; Uversky, 2019; Shmookler Reis et al., 2021). Examples of dysregulated IDPs include tau and Aβ_1–42_ in AD, TDP-43 in ALS and other diseases, and α-synuclein in PD (Irwin et al., 2013; Burre et al., 2018). We found many IDPs enriched in aggregates from human-AD hippocampus and in diverse *C. elegans* models of human neuropathic aggregation (Ayyadevara et al., 2015).

Not all IDPs are enriched in aggregates, and so we sought to identify properties that determine whether proteins are incorporated into aggregates or excluded from them. We utilized tools developed previously to predict protein disorder and aggregation propensity (Conchillo-Sole et al., 2007; Xue et al., 2010), but tailored our approach to allow us to infer which IDP properties favor or disfavor their entry into PD and AD aggregates. We combined 3 machine-learning methods and 49 predictors (several of which were scores from other machine-learning algorithms) to predict whether an IDP will enter into detergent-insoluble or detergent-soluble aggregates.

This strategy has the important benefit of providing insights into the most influential factors used by NN or SVM algorithms. Our NN predictions suggested that a combination of crucial physiochemical properties of a disordered protein are, at least in part, responsible for entry of a disordered protein into aggregates. Properties such as the abundance of basic or aromatic residues are predicted to be among the crucial factors in determining a disordered protein’s aggregation propensity (Table 3). Some of our predictions are supported by real-world examples, including tau, TDP-43 and α-synuclein (Deckert et al., 2016). We used principal component analysis to reduce the number of orthogonal inputs (“dimensionality”) for neural-network and SVM algorithms, and thus to define a minimal set of non-redundant determinants necessary to predict IDP aggregation. This approach was successful, in that restricting inputs to the first 3 PCA components only reduced the accuracy of SVM and NN by <5% (Table 4), enabling us to conclude that ***expected disorder*** is the most influential predictor for aggregation of specific proteins, followed by ***relative aggregate burden*** (i.e., the overall protein content of any of 5 aggregate subtypes). Disorder appears to be, by far, the most influential factor, enhanced somewhat by aggregate abundance. These predictions benefited only rather modestly from a variety of sequence-based determinants of aggregation propensity.

**FIGURE 6:**
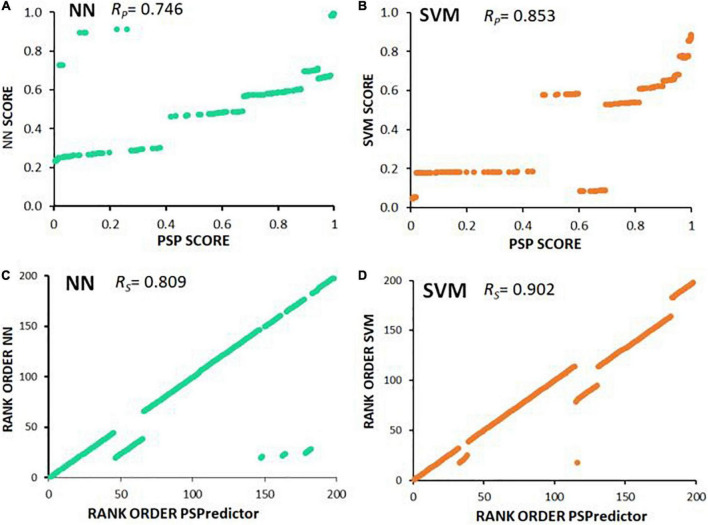
Scatter plots illustrate Spearman and Pearson correlations. **(A,B)** PSPredictor scores (Chu et al., 2022) (y axes) are plotted against aggregation propensities (x axes) predicted for 197 proteins, based on **(A)** neural network (NN), or **(B)** support vector machine (SVM). **(C,D)** Rank orders of PSPredictor scores (y axes) are plotted against rank orders of aggregation propensities (x axes) predicted for **(C)** neural network (NN), or **(D)** support vector machine (SVM). *R*_*P*_ is the Pearson (linear) correlation coefficient; *R*_*S*_ is the Spearman rank-order correlation coefficient.

It is intriguing that the predicted susceptibility of proteins to liquid-liquid phase separation (Deckert et al., 2016) correlated fairly well (*R*_*P*_ = 0.746) with neural-network prediction of entry into observed AD and PD aggregates, and a bit better (*R*_*P*_ = 0.853) with SVM predictions (Figure 6 and Supplementary Table 1). This suggests that the underlying “logic” employed by NN and SVM to predict aggregate inclusion, to some extent employs features also used by PSPredictor to assign likelihood of a liquid-liquid phase separation.

## Data availability statement

The raw data supporting the conclusions of this article will be made available by the authors, without undue reservation.

## Author contributions

Experiments were planned and interpreted by AG, MB, SA, and RJSR. Data were analyzed by AG and MB. The manuscript was written by AG, SA, and RJSR with input from MB on computational matters. All authors contributed to the article and approved the submitted version.

## Footnotes

1
https://disprot.org

2
http://www.pondr.com/

3
http://old.protein.bio.unipd.it/espritz/

4
http://bioinf.uab.es/aggrescan/

5
http://www.pkumdl.cn:8000/PSPredictor/

6
https://simlab.uams.edu/
