# Supplementary material for: Machine-learning analysis of intrinsically disordered proteins identifies key factors that contribute to neurodegeneration-related aggregation
Source: Front Aging Neurosci. 2022 Aug 3;14:938117. doi: 10.3389/fnagi.2022.938117 (PMC9382113; doi:10.3389/fnagi.2022.938117)
Supplement: Supplementary file 1 [file Data_Sheet_1.PDF]

Supplementary Table S1: AUC variation with NN parameter input choices

NN AUC	Number of hidden layers	Activation	Solver	Regularization	Maximal iterations
0.817	300	ReLu	SGD	0.0005	1370
0.808	200	ReLu	SGD	0.0005	5370
0.809	100	ReLu	SGD	0.0005	5370
0.816	100	Identity	SGD	0.0001	5370
0.817	200	Identity	Adam	0.0005	5370
0.816	300	Identity	Adam	0.0001	5370
0.816	100	Logistics	SGD	0.0005	5370
0.816	200	Logistics	Adam	0.0001	5370
0.816	300	Logistics	Adam	0.0005	5370
0.812	300	ReLu	Adam	0.0001	5370
0.803	200	ReLu	Adam	0.0001	4370
0.808	100	ReLu	Adam	0.0001	5370

Supplementary Table S2: AUC variation with SVM parameter input choices

The "optimal" parameter set is highlighted.

SVM AUC	Cost	Regression loss	Kernel	Numerical tolerance	Iteration limit
0.821	0.10	0.3	0.01	0.00010	100
0.819	0.10	0.4	0.01	0.00010	100
0.820	0.10	0.5	0.01	0.00010	100
0.819	0.10	0.10	0.01	0.00010	100
0.824	0.30	0.10	0.01	0.00010	200
0.825	0.40	0.10	0.01	0.00010	100
0.825	0.60	0.10	0.01	0.00010	100
0.817	0.10	0.10	Auto	0.00010	100
0.818	0.20	0.10	Auto	0.00010	100
0.821	0.30	0.10	Auto	0.00010	100
0.819	0.10	0.10	0.01	0.00011	100
0.821	0.30	0.10	0.01	0.00011	100
0.826	0.30	0.40	0.01	0.00011	100
0.823	0.30	0.50	0.01	0.00010	100
0.825	0.40	0.40	0.01	0.00010	150
0.823	0.40	0.40	0.01	0.00010	200
0.824	0.30	0.40	0.01	0.0010	200
0.822	0.20	0.40	0.01	0.0010	200
0.819	0.10	0.40	0.01	0.0010	200
0.823	0.80	0.90	0.01	0.0010	200

Supplementary Table S3: AUCs for four protein-partition categories

	Support Vector Machine (SVM)	Neural Network (NN)
**INSOL**	0.851	0.827
**BOTH**	0.934	0.894
**NOAGG**	0.800	0.780
**SOL**	0.637	0.563

Supplementary Table S4. PS Predictor Scores vs. NN (INSOL) and SVM (INSOL) Prediction Scores

	K	PSP Score	NN (INSOL)	SVM (INSOL)	Rank PSP	Rank NN	Rank SVM
Q2M2I8	AAK1_HUMAN	0.998	0.995	0.887	1	1	1
Q01546	K22O_HUMAN	0.998	0.995	0.886	2	2	2
P13645	K1C10_HUMAN	0.997	0.995	0.873	3	3	3
P23246	SFPQ_HUMAN	0.996	0.995	0.872	4	4	4
Q8N7X1	RMXL3_HUMAN	0.996	0.994	0.867	5	5	5
Q13151	ROA0_HUMAN	0.996	0.994	0.866	6	6	6
P35527	K1C9_HUMAN	0.996	0.994	0.866	7.5	7	7
P04264	K2C1_HUMAN	0.996	0.994	0.865	7.5	8	8
Q16629	SRSF7_HUMAN	0.996	0.994	0.865	9	9	9
P51116	FXR2_HUMAN	0.995	0.985	0.865	10	10	10
Q08170	SRSF4_HUMAN	0.994	0.984	0.863	11	11	11
Q92945	FUBP2_HUMAN	0.994	0.984	0.863	12	12	12
Q8NC51	PAIRB_HUMAN	0.993	0.984	0.861	13	13	13
Q7Z2K8	GRIN1_HUMAN	0.992	0.983	0.859	14	14	14
O14964	HGS_HUMAN	0.992	0.983	0.858	15	15	15
Q15517	CDSN_HUMAN	0.990	0.983	0.857	16	16	16
P16403	H12_HUMAN	0.989	0.983	0.856	17	17	17
O00401	WASL_HUMAN	0.988	0.983	0.779	18	18	21.5
Q12906	ILF3_HUMAN	0.987	0.983	0.778	19	19	23.5
Q96GY0	ZC21A_HUMAN	0.986	0.675	0.778	20	50	25.5
Q13263	TIF1B_HUMAN	0.983	0.673	0.777	21.5	51	27
P16402	H13_HUMAN	0.983	0.672	0.777	21.5	52	28
O95886	DLGP3_HUMAN	0.982	0.671	0.777	23	53	29
P29966	MARCS_HUMAN	0.981	0.671	0.777	24	54	30
Q5JU85	IQEC2_HUMAN	0.981	0.671	0.774	25	55	31
Q16186	ADRM1_HUMAN	0.980	0.670	0.773	26	56	32
Q9BUJ2	HNRL1_HUMAN	0.980	0.670	0.772	27	57	33
Q16204	CCDC6_HUMAN	0.977	0.670	0.772	28	58	34
Q02952	AKA12_HUMAN	0.977	0.670	0.771	29	59	35
Q99490	AGAP2_HUMAN	0.971	0.669	0.770	30	60	36
P43243	MATR3_HUMAN	0.969	0.669	0.770	31	61	37
P08779	K1C16_HUMAN	0.968	0.668	0.770	32	62	38
O60641	AP180_HUMAN	0.968	0.668	0.781	33	63	18
Q92522	H1X_HUMAN	0.967	0.667	0.780	34	64	19
Q3KQU3	MA7D1_HUMAN	0.965	0.667	0.780	35	65	20
Q63ZY3	KANK2_HUMAN	0.965	0.666	0.779	36	66	21.5
Q5SQI0	ATAT_HUMAN	0.961	0.665	0.778	37	67	23.5
Q8WXD9	CSKI1_HUMAN	0.957	0.665	0.778	38	68	25.5
Q9H3Q1	BORG4_HUMAN	0.954	0.663	0.685	39	69	39
Q9Y4F5	C170B_HUMAN	0.950	0.663	0.684	40	70	40
O75363	BCAS1_HUMAN	0.949	0.662	0.684	41	71	41
O43602	DCX_HUMAN	0.945	0.661	0.681	42	72	42
O75822	EIF3J_HUMAN	0.944	0.660	0.680	43	73	43
Q9C0H9	SRCN1_HUMAN	0.944	0.658	0.679	44	74	44
P19338	NUCL_HUMAN	0.941	0.658	0.679	45	75	45
Q9H4G0	E41L1_HUMAN	0.940	0.710	0.678	46	30	46
Q6NY19	KANK3_HUMAN	0.939	0.710	0.675	47	31	47
Q96SB3	NEB2_HUMAN	0.939	0.707	0.674	48	32	48
P54727	RD23B_HUMAN	0.939	0.705	0.673	49	33	49
Q9NZ56	FMN2_HUMAN	0.939	0.705	0.670	50.5	34	50

O75526	RMXL2_HUMAN	0.939	0.705	0.670	50.5	35	51
Q96D71	REPS1_HUMAN	0.936	0.703	0.663	52	36	52
O14490	DLGP1_HUMAN	0.936	0.703	0.660	53	37	53
Q53T59	H1BP3_HUMAN	0.933	0.703	0.660	54	38	54
O43491	E41L2_HUMAN	0.931	0.703	0.659	55	39	55
Q7Z6L0	PRRT2_HUMAN	0.929	0.702	0.658	56	40	56
Q9HBL0	TENS1_HUMAN	0.928	0.701	0.657	57	41	57
Q8NC96	NECP1_HUMAN	0.923	0.701	0.656	58	42	58
Q07157	ZO1_HUMAN	0.918	0.700	0.655	59	43	59
O43566	RGS14_HUMAN	0.910	0.699	0.654	60	44	60
O75122	CLAP2_HUMAN	0.903	0.699	0.652	61	45	61
P08572	CO4A2_HUMAN	0.898	0.699	0.652	62	46	62
Q8N111	CEND_HUMAN	0.897	0.697	0.624	63	47	63
P22528	SPR1B_HUMAN	0.897	0.697	0.624	64	48	64
Q6DN90	IQEC1_HUMAN	0.891	0.696	0.624	65	49	65
P09038	FGF2_HUMAN	0.880	0.606	0.623	66	76	66
Q9H1K0	RBNS5_HUMAN	0.879	0.604	0.623	67	77	67
P52272	HNRPM_HUMAN	0.868	0.603	0.623	68	78	68
Q9BXP5	SRRT_HUMAN	0.866	0.602	0.622	69	79	69
P84103	SRSF3_HUMAN	0.865	0.602	0.617	70	80	70
Q7Z4S6	KI21A_HUMAN	0.854	0.601	0.617	71	81	71
Q5JWF2	GNAS1_HUMAN	0.853	0.596	0.615	72	82	72
P02533	K1C14_HUMAN	0.839	0.596	0.614	73	83	73
Q9Y4G8	RPGF2_HUMAN	0.826	0.595	0.613	74	84	74
Q9UDT6	CLIP2_HUMAN	0.826	0.592	0.612	75	85	75
Q96AQ6	PBIP1_HUMAN	0.822	0.591	0.611	76	86	76
P39060	COIA1_HUMAN	0.821	0.590	0.611	77	87	77
P02452	CO1A1_HUMAN	0.814	0.590	0.611	78	88	78
Q14244	MAP7_HUMAN	0.812	0.589	0.541	79	89	95
Q9C0D0	PHAR1_HUMAN	0.800	0.588	0.540	80	90	96
P35326	SPR2A_HUMAN	0.797	0.588	0.540	81	91	97
Q14699	RFTN1_HUMAN	0.795	0.587	0.540	82	92	98
O60784	TOM1_HUMAN	0.794	0.586	0.540	83	93	99
Q14160	SCRIB_HUMAN	0.782	0.585	0.540	84	94	100
Q99961	SH3G1_HUMAN	0.777	0.584	0.539	85	95	101
O60610	DIAP1_HUMAN	0.774	0.583	0.538	86	96	102
Q9UDY2	ZO2_HUMAN	0.771	0.583	0.538	87	97	103
Q9NSK0	KLC4_HUMAN	0.767	0.583	0.537	88	98	104
P17931	LEG3_HUMAN	0.758	0.582	0.537	89	99	105
O15083	ERC2_HUMAN	0.746	0.576	0.537	90	100	106
P14678	RSMB_HUMAN	0.740	0.576	0.535	91	101	107
O14974	MYPT1_HUMAN	0.736	0.576	0.535	92	102	108
Q15599	NHRF2_HUMAN	0.726	0.575	0.534	93	103	109
P05387	RLA2_HUMAN	0.713	0.575	0.531	94	104	110
P46108	CRK_HUMAN	0.707	0.574	0.530	95	105	111
Q8IUX7	AEBP1_HUMAN	0.693	0.574	0.530	96	106	112
Q5JTV8	TOIP1_HUMAN	0.692	0.574	0.091	97	107	164
O75335	LIPA4_HUMAN	0.691	0.573	0.091	98	108	165
Q9H9H4	VP37B_HUMAN	0.684	0.572	0.090	99	109	166
O60282	KIF5C_HUMAN	0.683	0.570	0.090	100	110	167
Q9P1Y5	CAMP3_HUMAN	0.681	0.569	0.089	101	111	168

Q9BTT0	AN32E_HUMAN	0.679	0.569	0.089	102	112	169
Q8WXE0	CSKI2_HUMAN	0.677	0.569	0.089	103	113	170
P09493	TPM1_HUMAN	0.676	0.568	0.089	104	114	171
Q9BYB0	SHAN3_HUMAN	0.672	0.491	0.089	105	115	172
O15400	STX7_HUMAN	0.659	0.489	0.088	106	116	173
Q9UKE5	TNIK_HUMAN	0.657	0.489	0.088	107	117	174.5
Q8NCB2	CAMKV_HUMAN	0.651	0.488	0.088	108	118	174.5
Q9UL54	TAOK2_HUMAN	0.639	0.488	0.087	109	119	176
Q13136	LIPA1_HUMAN	0.614	0.487	0.087	110	120	177
Q15233	NONO_HUMAN	0.609	0.487	0.087	111	121	178
Q16799	RTN1_HUMAN	0.604	0.487	0.087	112	122	179
Q08174	PCDH1_HUMAN	0.603	0.486	0.086	113	123	180
Q99456	K1C12_HUMAN	0.603	0.485	0.085	114	124	181
Q7Z6B0	CCD91_HUMAN	0.594	0.484	0.586	115	125	79
O95678	K2C75_HUMAN	0.585	0.484	0.585	116	126	80
Q96QR8	PURB_HUMAN	0.584	0.481	0.584	117	127	81
P22455	FGFR4_HUMAN	0.574	0.481	0.584	118	128	82
Q92688	AN32B_HUMAN	0.568	0.479	0.584	119	129	83
O76070	SYUG_HUMAN	0.568	0.478	0.584	120	130	84
Q1KMD3	HNRL2_HUMAN	0.562	0.478	0.583	121	131	85
Q9ULE4	F184B_HUMAN	0.558	0.477	0.583	122	132	86
P51513	NOVA1_HUMAN	0.553	0.476	0.582	123	133	87
Q9NQC3	RTN4_HUMAN	0.552	0.476	0.582	124	134	88
Q9BQI7	PSD2_HUMAN	0.522	0.475	0.582	125	135	89
Q14203	DCTN1_HUMAN	0.515	0.473	0.581	126	136	90
Q9H8Y8	GORS2_HUMAN	0.477	0.471	0.581	127	137	91
Q92764	KRT35_HUMAN	0.474	0.471	0.580	128	138	92
Q9UJU6	DBNL_HUMAN	0.471	0.469	0.580	129	139	93
O75937	DNJC8_HUMAN	0.469	0.469	0.580	130	140	94
Q8N1G4	LRC47_HUMAN	0.433	0.468	0.186	131	141	113
Q96S90	LYSM1_HUMAN	0.432	0.466	0.186	132	142	114
Q00341	VIGLN_HUMAN	0.417	0.464	0.186	133	143	115
Q05682	CALD1_HUMAN	0.416	0.463	0.186	134	144	116
O14562	UBFD1_HUMAN	0.377	0.304	0.186	135	145	117
P05787	K2C8_HUMAN	0.375	0.303	0.186	136	146	118
P49593	PPM1F_HUMAN	0.365	0.301	0.186	137	147	119
P08729	K2C7_HUMAN	0.350	0.300	0.185	138	148	120
Q99963	SH3G3_HUMAN	0.316	0.296	0.185	139	149	121
Q4V328	GRAP1_HUMAN	0.311	0.296	0.185	140	150	122
O76011	KRT34_HUMAN	0.301	0.292	0.185	141	151	123
Q9H6N6	MYH16_HUMAN	0.293	0.291	0.185	142	152	124
Q14152	EIF3A_HUMAN	0.289	0.290	0.185	143	153	125
Q9NQG5	RPR1B_HUMAN	0.289	0.290	0.185	144	154	126
O00233	PSMD9_HUMAN	0.275	0.289	0.185	145	155	127
Q6ZVM7	TM1L2_HUMAN	0.273	0.289	0.185	146	156	128
Q8IUD2	RB6I2_HUMAN	0.260	0.914	0.185	147	20	129
P54725	RD23A_HUMAN	0.222	0.914	0.184	148	21	130
Q15276	RABE1_HUMAN	0.197	0.278	0.184	149	157	131
Q9NVI7	ATD3A_HUMAN	0.173	0.277	0.184	150	158	132
P16949	STMN1_HUMAN	0.166	0.276	0.184	151	159	133
O60749	SNX2_HUMAN	0.166	0.276	0.184	152	160	134

Q9H444	CHM4B_HUMAN	0.162	0.276	0.184	153	161	135
Q9NYB0	TE2IP_HUMAN	0.157	0.275	0.184	154	162	136
P78318	IGBP1_HUMAN	0.156	0.274	0.184	155	163	137
Q86WG3	ATCAY_HUMAN	0.147	0.273	0.184	156	164	138
Q96A00	PP14A_HUMAN	0.139	0.272	0.183	157	165	139
Q9GZM8	NDEL1_HUMAN	0.135	0.271	0.183	158	166	140
P83731	RL24_HUMAN	0.133	0.270	0.183	159	167	141
P62805	H4_HUMAN	0.123	0.268	0.183	160	168	142
Q9H019	MFR1L_HUMAN	0.113	0.898	0.182	161	22	143
O95989	NUDT3_HUMAN	0.105	0.897	0.182	162	23	144
P30533	AMRP_HUMAN	0.090	0.896	0.182	163	24	145
P50479	PDLI4_HUMAN	0.090	0.264	0.182	164	169	146
P35900	K1C20_HUMAN	0.086	0.263	0.182	165	170	147
P62851	RS25_HUMAN	0.069	0.263	0.181	166	171	148
Q6ZVX7	FBX50_HUMAN	0.064	0.262	0.181	167	172	149
O76013	KRT36_HUMAN	0.061	0.260	0.181	168	173	150
P54105	ICLN_HUMAN	0.055	0.260	0.181	169	174	151
Q9UHG2	PCSK1_HUMAN	0.046	0.259	0.181	170	175	152
P01877	IGHA2_HUMAN	0.044	0.259	0.181	171	176	153
P49207	RL34_HUMAN	0.037	0.258	0.181	172	177	154
Q9Y5S9	RBM8A_HUMAN	0.035	0.258	0.180	173	178	155
P55036	PSMD4_HUMAN	0.035	0.256	0.180	174	179	156
Q9BRT3	MIEN1_HUMAN	0.031	0.256	0.180	175	180	157
P60866	RS20_HUMAN	0.029	0.255	0.180	176	181	158
O95670	VATG2_HUMAN	0.029	0.730	0.180	177	25	159
Q9HAP6	LIN7B_HUMAN	0.026	0.730	0.180	178	26	160
P62854	RS26_HUMAN	0.022	0.729	0.179	179	27	161
P62899	RL31_HUMAN	0.021	0.729	0.179	180	28	162
Q9UNX3	RL26L_HUMAN	0.018	0.728	0.179	181	29	163
Q7LBR1	CHM1B_HUMAN	0.017	0.250	0.057	182	182	182.5
P04792	HSPB1_HUMAN	0.015	0.250	0.057	183	183	182.5
Q9UHV9	PFD2_HUMAN	0.015	0.247	0.056	184	184	184
P63208	SKP1_HUMAN	0.012	0.246	0.056	185	185	185
O60262	GBG7_HUMAN	0.011	0.244	0.055	186	186	186
Q9NUV9	GIMA4_HUMAN	0.010	0.244	0.055	187	187	187
O95292	VAPB_HUMAN	0.010	0.243	0.055	188	188	188
Q86Y82	STX12_HUMAN	0.009	0.242	0.053	189	189	189
Q6PUV4	CPLX2_HUMAN	0.009	0.242	0.052	190	190	190
P07919	QCR6_HUMAN	0.008	0.242	0.050	191	191	191
P62269	RS18_HUMAN	0.007	0.238	0.050	192	192	192
P63027	VAMP2_HUMAN	0.007	0.238	0.050	193	193	193
O00161	SNP23_HUMAN	0.006	0.237	0.049	194	194	194
Q8N128	F177A_HUMAN	0.005	0.237	0.049	195	195	195
Q9Y5K8	VATD_HUMAN	0.004	0.236	0.049	196	196	196
O43752	STX6_HUMAN	0.003	0.235	0.047	197	197	197
**Correlation Coefficient vs. PSPredictor:**			**0.746**	**0.853**		**0.809**	**0.902**
